# A Rare Case of Diffuse Hemangiomatosis of the Spleen with Splenic Rupture following Aortic Valve Replacement

**DOI:** 10.1155/2017/9164749

**Published:** 2017-01-12

**Authors:** F. Capilli, M. Weinbeck, M. Czerny, M. Siepe, T. Krauss

**Affiliations:** ^1^Department of Radiology, Section of Cardiovascular Radiology, University Hospital Freiburg, Freiburg, Germany; ^2^Department of Cardiovascular Surgery, Heart Center Freiburg, Bad Krozingen, Germany

## Abstract

In this paper we present a rare case of splenic rupture that occurred after an open aortic valve replacement in a male patient with hemangiomatosis of the spleen and the liver. The patient was treated with an emergency splenectomy. He showed no other sings of associated systemic disorder, such as Klippel-trénaunay syndrome or Proteus syndrome.

## 1. Introduction

Splenic hemangiomas are the most common primary neoplasm of the spleen. Usually they are asymptomatic incidental findings and can easily be diagnosed with computed tomography (CT) or magnetic resonance imaging (MRI), due to their typical contrast enhancement. As benign tumours, splenic hemangiomas usually do not require any treatment [1, 2]. Rarely, especially if localized under the splenic capsule, splenic hemangiomas can cause subcapsular hematomas, complete splenic rupture, and consequently severe internal bleeding. A diffuse splenic hemangiomatosis, which forms multiple tumours within the whole spleen, is very rare and can cause spontaneous splenic rupture [3].

## 2. Case Presentation

A 68-year-old man underwent an open aortic valve replacement because of severe aortic valve stenosis. The day after the open-heart-operation a revision thoracotomy was needed, because of postoperative acute thoracic bleeding, and during the procedure a cardiopulmonary resuscitation was performed.

Three days after first surgery the patient became again hemodynamically unstable and presented signs of hemorrhagic shock.

Sonography demonstrated an enlargement of the spleen and free fluid in the peritoneal space. Pericardial and pleural effusions could be excluded.

**Figure 1:**
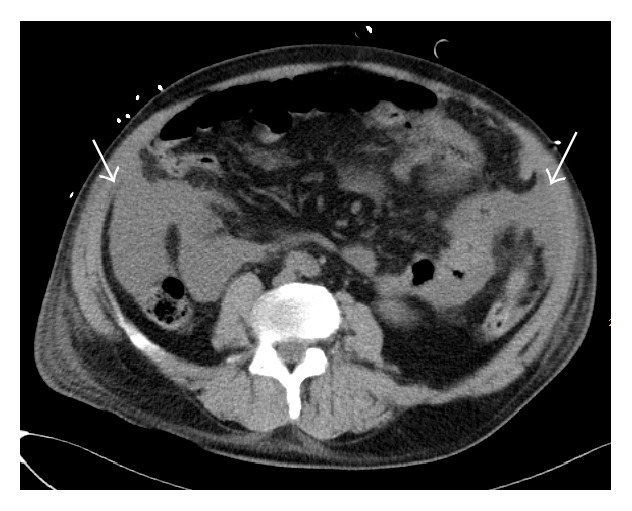
Nonenhanced CT scan of the abdomen shows blood in the peritoneal space (white arrows).

**Figure 2:**
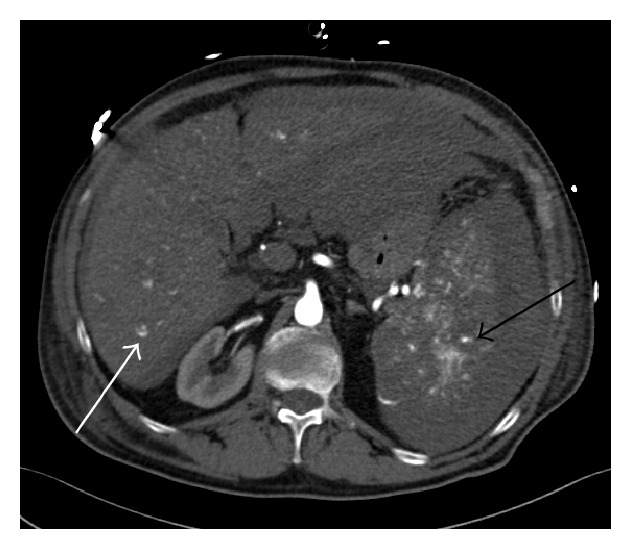
Arterial phase of contrast-enhanced CT scan depicts multiple hypervascular lesions in the spleen (black arrow) and liver (white arrow).

**Figure 3:**
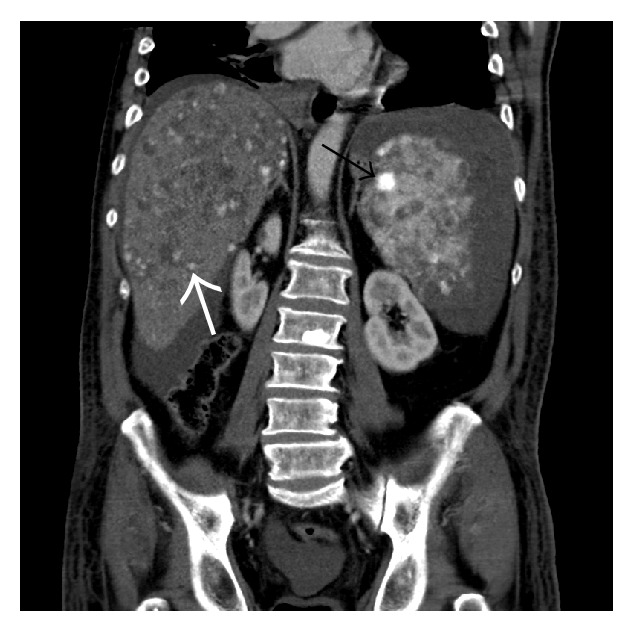
Venous phase: multiple hepatic (white arrow) and splenic (black arrow) lesions.

**Figure 4:**
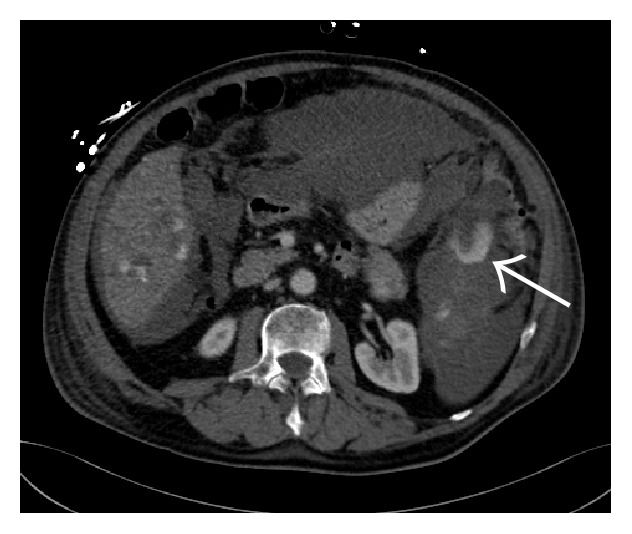
Venous phase: extravasation of contrast agent suggesting perisplenic bleeding (white arrow).

A multiphasic contrast-enhanced CT scan of the abdomen confirmed a large amount of intraperitoneal blood and an enlargement of the spleen caused by a subcapsular hematoma (Figure 1). The contrast-enhanced images of the CT scan (arterial and venous phase) demonstrated a heterogenous contrast enhancement of the spleen and of the liver with several hypervascular focal lesions in both organs, which then became more evident in the portal-venous phase of the CT scan (Figures 2 and 3). An acute perisplenic bleeding could also be depicted, suggesting a splenic capsular tear (Figure 4). There was no evidence of rib fracture.

The patient underwent an emergency splenectomy with perioperative transfusion of 11 erythrocytes concentrates. During early postoperative care the patient was hemodynamically stable.

In the days following the abdominal procedure the patient suffered from intermittent abdominal bleeding and underwent recurrent surgical treatment. 10 days after the initial procedure the patient died due to the development of a multiorganic failure.

The immunohistochemical analysis revealed the endothelial cells to be strong CD34-positive and also to express focally CD31. Factor8 was positive on the membranes. Many macrophages were immunostained for CD68, while the endothelial cells remained mainly negative. CD8 was not expressed in the most part of the vessels. The growth fraction was circa 15%. The pathologist reported a “diffuse, mixed cavernous and capillary splenic hemangioma, affecting the entire spleen, with splenic capsular rupture.” Although no biopsy of the liver was performed, the CT-appearance is highly suggestive of hepatic involvement, leading to the diagnosis of a hemangiomatosis of the spleen and liver.

## 3. Discussion

The role of contrast-enhanced CT scan is paramount in depicting hypervascular splenic tumours, identifying typical malignant features, like aggressive growth with infiltration of near structures and area of necrosis of the angiosarcomas, and diagnosing possible complication [4]. Because of the often similar unspecific appearance of primary splenic lesions, such as littoral cell angiomas, lymphangiomas, hamartomas, peliosis, hemangiopericytomas, and hemangioendotheliomas as well as some hemangiomas themselves, a pathological and immunohistochemical analysis is in many cases needed to secure the diagnosis [5].

Splenic hemangiomatosis presents as multifocal or diffuse localization of splenic hemangiomas [6–9]. This condition is very rare and is usually described in association with other systemic disorders such as Klippel-trénaunay syndrome or Proteus syndrome [10, 11]. Possible clinical presentations include also Kasabach-Merritt syndrome, which consists in a consumptive coagulopathy due to large vascular tumours [12]. Spontaneous splenic ruptures are usually reported as a complication of infectious diseases, hemopathies, or storage diseases associated with splenomegaly [13].

Delayed splenic injury is a very rare complication of CPR [14] and we see no clear temporal correlation between the CPR and the acute abdominal hemorrhage in our patient. It can be however supposed that extensive parenchymal damage, due to the hemangiomatosis, increased the vulnerability of the organ, exposing it to rupture even without a relevant trauma. We suggest that the parenchymal disease of the spleen, the hemodynamic stress of an open-heart operation, and an anticoagulation therapy may have caused a delayed splenic rupture in our patient after CPR.

## 4. Conclusion

This case illustrates a delayed splenic rupture in a patient with hemangiomatosis of the spleen and the liver after open aortic valve replacement and CPR. Although very rare, this condition can possibly cause life-threatening complications after CPR.

## References

[1] Steininger H., Pfofe D., Marquardt L., Sauer H., Markwat R. (2004). Isolated diffuse hemangiomatosis of the spleen: case report and review of literature. *Pathology Research and Practice*.

[2] Ambrosio M. R., Rocca B. J., Di Mari N., Ambrosio A., Lazzi S. (2012). Multifocal capillary hemangioma (hemangiomatosis) of the spleen. *Tumori*.

[3] Karakayali F., Basaran C., Soy E. A. (2010). Spontaneous spleen rupture and rectus sheath hematoma in a patient with Klippel-Trenaunay syndrome: report of a case. *Surgery Today*.

[4] Abbott R. M., Levy A. D., Aguilera N. S., Gorospe L., Thompson W. M. (2004). Primary vascular neoplasms of the spleen: radiologic-pathologic correlation. *Radiographics*.

[5] Fotiadis C. I., Georgopoulos I., Stoidis C., Patapis P. (2009). Primary tumors of the spleen. *International Journal of Biomedical Science*.

[6] Makino I., Tajima H., Kitagawa H., Nakagawara H., Ohta T. (2014). A rare case of hemangiomatosis of the spleen and intrapancreatic accessory spleen. *Abdominal Imaging*.

[7] Moss C. N., Van Dyke J. A., Koehler R. E., Smedberg C. T. (1986). Multiple cavernous hemangiomas of the spleen: CT findings. *Journal of Computer Assisted Tomography*.

[8] Latifi H. R., Siegel M. J. (1992). Diffuse neonatal hemangiomatosis: CT findings in an adult. *Journal of Computer Assisted Tomography*.

[9] Nakano Y., Fujisaki H., Ishiguro T. (2015). Isolated diffuse hemangiomatosis of the spleen with disseminated intravascular coagulation: successful treatment with embolization and splenectomy. *Journal of Pediatrics*.

[10] Dekeyzer S., Houthoofd B., De Potter A., Van Bockstal M., Smeets P., Vogelaers D. (2013). Hemangiomatosis of the spleen in a patient with Klippel-trénaunay syndrome. *JBR-BTR*.

[11] Wang Z., Yu Z., Su Y. (2007). Kasabach-Merritt syndrome caused by giant hemangiomas of the spleen in patients with Proteus syndrome. *Blood Coagulation and Fibrinolysis*.

[12] Tang J.-Y., Chen J., Pan C., Yin M.-Z., Zhu M. (2008). Diffuse cavernous hemangioma of the spleen with Kasabach-Merritt syndrome misdiagnosed as idiopathic thrombocytopenia in a child. *World Journal of Pediatrics*.

[13] Skok P., Knehtl M., Ćeranić D., Glumbić L. (2009). Splenic rupture in systemic amyloidosis—case presentation and review of the literature. *Zeitschrift fur Gastroenterologie*.

[14] Pestaner J. P., Smialek J. E. (2002). Splenic rupture following cardiopulmonary resuscitation. *Resuscitation*.

